# **Advances in understanding the functions and regulatory factors of secondary metabolites in** ***Reynoutria japonica***

**DOI:** 10.1080/15592324.2025.2564957

**Published:** 2025-10-04

**Authors:** Yanli Zhang, Jiaxing Huang, Jianhui Jia, Jiayan Jiang, Xinyan Ma, Lili Zhao, Yunrong Jing

**Affiliations:** School of Life Science and Technology, Mudanjiang Normal University, Mudanjiang, China

**Keywords:** *Reynoutria japonica*, biosynthetic mechanisms, secondary metabolites, pharmacological functions, influencing factors

## Abstract

*Reynoutria japonica*, a perennial herb of the *Polygonaceae* family, is a traditional Chinese medicinal plant known for its diverse pharmacological activities and broad applications in medicine, agriculture, and related fields. This review explores the functions and regulatory mechanisms of its secondary metabolites by summarizing their types, bioactivities, and biosynthetic regulation. Additionally, it examines how factors such as plant age, medicinal organ, soil composition, and cultivation conditions influence the secondary metabolite profile. These insights support the clinical application and industrial development of *R. japonica*.

## 1. Introduction

*Reynoutria japonica*, a perennial herb belonging to the *Polygonaceae* family and *Reynoutria* genus, is widely used in traditional Chinese medicine. While young stems are edible, the roots hold significant medicinal value and are known for their therapeutic properties, including clearing heat, detoxifying, eliminating dampness, promoting blood circulation, and regulating meridians. It is predominantly distributed in provinces such as Shaanxi, as well as central and south-central regions of China.^1^^,^^2^

Primary metabolites, such as sugars, amino acids, and lipids, are essential for basic plant growth, development, and reproduction. In contrast, secondary metabolites are a class of compounds synthesized during secondary metabolic processes in plants. They play vital roles in plant growth, environmental adaptation, and defense responses.^3^ *R. japonica* produces a wide range of secondary metabolites, notably flavonoids, phenolic acids, and anthraquinones. Key compounds such as resveratrol (3,5,4'-trihydroxy-trans-stilbene), polydatin (resveratrol-3-O-*β*-D-glucoside), and emodin (1,3,8-trihydroxy-6-methylanthraquinone) exhibit anti-inflammatory, antioxidant, antitumor, and immunomodulatory activities, with resveratrol and polydatin showing anti-inflammatory and antioxidant effects in preclinical models,^4^ emodin demonstrating antitumor activity,^5^ and all three contributing to immunomodulatory effects.^4^ These compounds have also shown therapeutic potential in treating gouty arthritis,^6^ protecting liver function,^7^ and regulating blood glucose levels.^8^^,^^9^ These pharmacological properties, supported by specific studies, highlight the promising clinical applications of *R. japonica*. However, limitations such as resveratrol’s low oral bioavailability because of rapid metabolism and poor solubility restrict its clinical efficacy, necessitating further formulation strategies such as nanoencapsulation and clinical trials to validate these effects.

The diversity and abundance of its secondary metabolites are influenced by various internal factors, such as plant age and specific medicinal organs (e.g., roots versus stems), and external factors, including soil nutrient composition (notably nitrogen and phosphorus), water availability, and cultivation conditions like light exposure. These factors limit the consistent expression of its medicinal potential and industrial scalability. This review provides a comprehensive overview of the types, biological functions, biosynthetic mechanisms, and influencing factors of *R. japonica* secondary metabolites, aiming to support its clinical use and inform the development of novel therapeutic agents.

To ensure a comprehensive research on the secondary metabolites of *R. japonica*, a systematic literature search was conducted using multiple databases: PubMed, Web of Science, and China National Knowledge Infrastructure (CNKI). These databases were selected to capture both international and Chinese-language studies, reflecting the prominence of plants in traditional Chinese medicine (TCM) and global phytochemical research. The search employed a combination of keywords, including secondary metabolites, *R. japonica*, biosynthetic mechanisms, pharmacological functions of *R. japonica*, influencing factors of secondary metabolites, etc.

## 2. Types of secondary metabolites of *R. japonica*

The secondary metabolites of *R. japonica* are of considerable importance in both medicine and biological research. In the pharmaceutical field, they serve as valuable natural sources for drug development, while in plant biology, they provide essential materials for studying physiological functions and ecological interactions.

*R. japonica* contains a rich and diverse profile of biochemical compounds. Based on their chemical structures, previous studies^5,10–15^ have categorized these secondary metabolites into several major groups, including quinones, stilbenes, and flavonoids. Key representatives of these classes include emodin^10^and chrysophanol (1,8-dihydroxy-3-methylanthraquinone),^16^ which are quinones; resveratrol (3,5,4'-trihydroxy-trans-stilbene)^4^ and polydatin (resveratrol-3-O-*β*-D-glucoside), which belong to the stilbene class; and quercetin (3,3',4',5,7-pentahydroxyflavone),^17^ a typical flavonoid (Table 1).

**Table 1.** Types of secondary metabolites of ***R. japonica.***

Number	Chemical structure	Name of secondary metabolite	Distribution site	Reported bioactivities	References
1	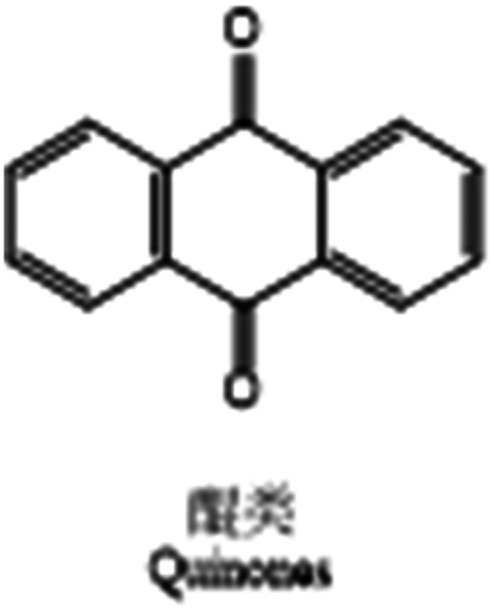	*Rheumatoid*	*Root, rhizome*	Laxative (activates intestinal Cl⁻ channels), anti-inflammatory (COX-2 inhibition), antibacterial (vs *S. aureus*, MIC = 50 μg/mL)	[18,19]
2		*Rheumic acid*	*Root*	Antifungal (inhibits *Candida albicans* biofilm formation)	[18]
3		*Rheumol*	*Root*	Weak cholagogue effect (↑ bile secretion by 18% in rats, 50 mg/kg)	[20]
4		*Rheumexigenin methyl ether*	*Root, rhizome*	Cytotoxic (inhibits >60% HT-29 colon cancer cells at 100 uM)	[18]
5		*Rheumannin 8-methyl ether*	*Root, rhizome*	Antiplatelet aggregation (inhibits ADP-induced platelet aggregation, IC₅₀ = 42 μM)	[21]
6		*1-O-β-D-glucoside of rhubarbine methyl ether*	*Root*	Laxative synergist (enhances rhein-induced colonic contraction by 1.8-fold)	[22]
7		*1-O-β-D-glucoside of rhubarbine*	*Root*	Laxative synergist (enhances rhein-induced colonic contraction by 1.8-fold)	[22]
8	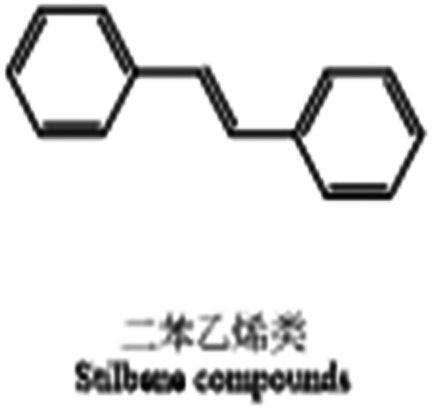	*cis-Resveratrol diphenyl*	*Root, rhizome*	Antioxidant, anti-inflammatory (inhibits TNF-a, IL-6 release)	[19]
9		*trans-Resveratrol diphenyl*	*Root, rhizome*	Cardioprotective (↓ myocardial infarction area, ↑ SOD activity), antifibrotic (inhibits TGF-β1 pathway)	[19]
10		*cis-Resveratrol diphenyl*	*Root, rhizome*	Bioactivity similar to No.8 (cis-isomer)	[19]
11		*trans-Resveratrol diphenyl*	*Root, rhizome*	Bioactivity similar to No.9 (trans-isomer)	[19]
12		*cis-Resveratrol-4'-O-glucoside diphenylene*	*Root, rhizome*	Hepatoprotective (↓ liver fibrosis marker HyP), crosses blood-brain barrier	[23]
13		*trans-Resveratrol-4'-O-glucoside diphenylene*	*Root, rhizome*	Neuroprotective (↑ Nrf2 pathway, ↓ cerebral infarction volume), antifibrotic (↓ PLA2/TGF-β1)	[19]
14		*Resveratrol-4-O-D-(2'-galloyl)glucopyranoside diphenylene*	*Root*	Inhibits the formation of advanced glycation end products (AGEs) with an IC₅₀ of 35 μM (compared to the positive control drug aminoguanidine, IC₅₀ = 42 μM)	[24]
15		*Resveratrol-4-O-D-(6'-galloyl)glucopyranoside diphenylene*	*Root*	Antiviral (inhibits influenza A virus replication, EC₅₀ = 7.8 μM)	[24]
16		*trans-Resveratrol-3-O-β-D-glucopyranoside-6″-sulfate diphenylene*	*Root*	Antioxidant (ROS scavenging), metabolic regulation (intestinal hydrolysis to aglycone)	[23,25]
17		*trans-Resveratrol-3-O-β-D-glucoside-4″-diphenyl sulfate*	*Root*	Core activities: • Anticancer (induces apoptosis in hepatoma cells, IC₅₀ = 28 μM) • Antioxidant (superior ROS scavenging vs aglycone) • Metabolic regulation (enhanced bioavailability via intestinal hydrolysis)	[23,25,26]
18		*trans-Resveratrol-3-O-β-D-glucoside-2″-diphenyl sulfate*	*Root*		
19		*trans-Resveratrol-3-O-β-D-glucoside-3″-diphenyl sulfate*	*Root*		
20		*trans-resveratrol-3-O-β-D-glucoside-5-sulfate diphenylene*	*Root*		
21		*cis-Resveratrol-3-O-β-D-glucoside-6″-diphenyl sulfate*	*Root*		
22		*cis-Resveratrol-3-O-β-D-glucoside-4″-diphenyl sulfate*	*Root*		
23		*cis-Resveratrol-3-O-β-D-glucoside-3″-diphenyl sulfate*	*Root*		
24		*cis-Resveratrol-3-O-β-D-glucoside-2″-diphenyl sulfate*	*Root*		
25		*cis-Resveratrol-3-O-β-D-glucoside-5-diphenyl sulfate*	*Root*		
26	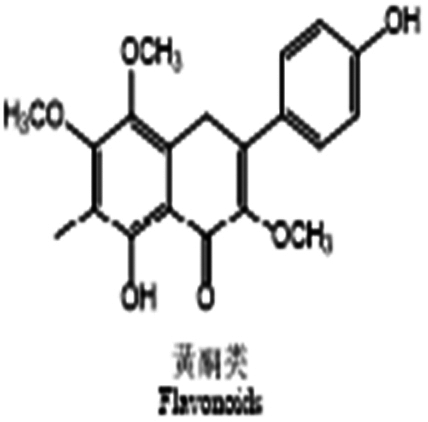	*Quercetin*	*Root, rhizome*	Antioxidant (DPPH EC₅₀ = 2.1 μM), anti-inflammatory (NF-κB inhibition)	[19]
27		*Quercetin-3-O-arabinoside*	*Root*	Vasoprotective (↑ endothelial NO synthesis), antidiabetic (*α*-glucosidase inhibition, IC₅₀ = 15 μM)	[19]
28		*Quercetin 3-O-rhamnoside*	*Root*	Anti-inflammatory:（Inhibits the NF-kB pathway and reduces the release of TNF-a and IL-6)	[27]
29		*Quercetin 3-O-galactoside*	*Root*	Anti-inflammatory:（Inhibits the NF-kB pathway and reduces the release of TNF-a and IL-6)	[27]
30		*Quercetin 3-O-glucoside*	*Root*	High bioavailability (38% intestinal absorption), systemic antioxidant	[19]
31		*Quercetin-3-xyloside*	*Leaf*	UV-protective (↓ UVB-induced keratinocyte apoptosis)	[19]

## 3. Pharmacological effects of secondary metabolites of *R. japonica*

### 3.1. Treatment of gouty arthritis

Gout is a form of inflammatory arthritis caused by excessive deposition of uric acid crystals, which affects approximately 1%–2% of adults in developed countries and poses a significant clinical burden due to recurrent pain, joint damage, and associated comorbidities.^28^ *R. japonica* is widely used in traditional medicine for the prevention and treatment of gout, owing to its properties of clearing heat, dispelling blood stasis, and relieving pain. The therapeutic potential of *R. japonica* and its secondary metabolites in managing gout-related conditions has been extensively studied. Clinical trials are needed to confirm these effects and address polydatin’s bioavailability, which may be limited by its glycosylated structure.

For instance, Ma and colleagues^29^ employed a mouse model of acute gouty arthritis induced by monosodium urate (MSU) injection into the ankle joint. They found that polydatin significantly inhibited the expression of inflammatory markers such as IL-6, TNF-*α*, IL-1β, as well as the protein and mRNA levels of Caspase-1, NLRP3, and ASC in synovial tissues. These findings suggest that polydatin can effectively intervene in the inflammatory progression of acute gouty arthritis.

Given that gout pathogenesis is closely associated with elevated uric acid levels and systemic inflammation, Xu Wenjing et al.^30^ utilized an MSU-induced injury model in HK-2 cells combined with the ABCG2 inhibitor Ko143. These results showed that polydatin could reduce uric acid accumulation by modulating urate transporter activity. Further studies using LPS + MSU-induced inflammation models in THP-1 cells, along with the NLRP3 inflammasome inhibitor MCC950 and the NF-kB pathway inhibitor QNZ, confirmed that polydatin exerts anti-inflammatory effects through the inhibition of the NLRP3 inflammasome and NF-kB signaling pathway.

In addition, Cheng Jing^31^ established an acute gouty arthritis rat model and administered *R. japonica* extract post-induction. The treatment markedly reduced joint swelling, as assessed by caliper measurements of joint diameter and histological scoring using hematoxylin–eosin (H&E) staining to evaluate synovial inflammation, further supporting its therapeutic efficacy (Table 2). These findings, which are consistent with the anti-inflammatory effects of *R. japonica* extract studies,^29^^,^^30^ highlighting polydatin’s potential in alleviating gouty arthritis symptoms through urate regulation and inflammation suppression.

**Table 2.** Pharmacology study summary.

Compound	Disease model	Dose	Outcomes	Mechanism of action	Limitation(s)	References
Polydatin	MSU-induced acute gouty arthritis in mice	Not specified	Inhibited IL-6, TNF-*α*, IL-1β; reduced protein/mRNA expression of Caspase-1, NLRP3, ASC in synovial tissues	Suppression of NLRP3 inflammasome activation	Low bioavailability due to glycosylated structure	
Polydatin	MSU-induced injury in HK-2 cells	Not specified	Reduced uric acid accumulation	Modulation of urate transporter activity	Lack of clinical validation	[30]
Polydatin	LPS + MSU-induced inflammation in THP-1 cells	Not specified	Significantly attenuated inflammation	Inhibition of NLRP3 inflammasome and NF-κB signaling pathway	Limitations of *in vitro* models	[30]
Polydatin	MSU-induced acute gouty arthritis in rats	Not specified	Reduced joint swelling; improved synovitis (confirmed by H&E staining)	Combined anti-inflammatory and urate-regulating effects	Active constituents not quantitatively analyzed	[31]
Polydatin derivative	SGLT2 inhibitory activity assay	Molecular level	Designed a novel SGLT2 inhibitor	Stilbene scaffold serving as aglycone core for C-aryl glycoside-type SGLT2 inhibitors	Lack of *in vivo* validation	[9]
*Polygonum cuspidatum Extract (containing Polygonum cuspidatum polysaccharide, polydatin, and other phenolic compounds, etc.)*	Type 2 diabetic rat model	Graded doses	Reduced FFA, TC, TG, LDL-C; Increased HDL-C	Regulation of lipid metabolism (enhanced fatty acid oxidation)	Specific active constituents not isolated	[32]
*R. japonica* polysaccharides	Diabetic rat model	Not specified	Significantly improved glucose tolerance (*P* < 0.05), comparable to metformin (*P* > 0.05)	Not elucidated	Mechanism of action not clarified	[33]
*R. japonica* aqueous decoction	CCl₄-induced liver injury model	Not specified	Reduced serum ALT/AST; Enhanced SOD/GSH-Px activity; decreased MDA	Augmentation of endogenous antioxidant defense systems	Short-term effects assessed	[9]*
Polydatin	Atherosclerotic rat model	Medium/high doses	Reduced TC/TG/LDL-C; Increased HDL-C; decreased hepatic cholesterol accumulation (confirmed by Oil Red O staining)	Modulation of lipid metabolism pathways	Low oral bioavailability	[34]

### 3.2. Glucose regulation

Type 2 diabetes mellitus, accounting for more than 90% of all diabetes cases, is primarily caused by impaired insulin secretion, insulin resistance, or a combination of both. Sodium-glucose cotransporter 2 (SGLT2) inhibitors have emerged as a key therapeutic strategy for managing type 2 diabetes, and polydatin, a key component of *R. japonica*, has shown promise in this area. Shi Yongheng^35^ reported that the stilbene structure of polydatin closely resembles that of vildagliptin, a known SGLT2 inhibitor. By integrating the stilbene scaffold of polydatin with the pharmacophoric features of vildagliptin, a novel SGLT2 inhibitor was designed from natural product origins.^35^ The stilbene backbone of polydatin can serve as the aglycone core of potent C-aryl glycoside-type SGLT2 inhibitors, providing a promising foundation for the development of new antidiabetic agents. This design has been supported by in vitro assays demonstrating inhibitory activity against sodium-glucose cotransporter 2 (SGLT2), confirming its potential as an antidiabetic agent.^35^ However, further in vivo studies and molecular docking analyses are recommended to fully validate its efficacy and mechanism of action. These findings highlight the therapeutic potential of *R. japonica*-derived compounds in blood glucose regulation.

Zhao et al.^32^ conducted a study investigating the direct antidiabetic effects of *R. japonica* extract using a rat model of type 2 diabetes. The experimental groups included a normal control, disease model, various doses of *R. japonica* extract, and a treatment group receiving XiaoKeJiangTang capsules, a traditional Chinese medicine formulation for glycemic control that contains active constituents such as berberine and other herbal extracts that are likely to increase insulin sensitivity and reduce lipid levels.^36^ Compared to the normal group, the diabetic model rats exhibited significantly elevated levels of free fatty acids FFA total cholesterol (TC), triglycerides TG and low-density lipoprotein cholesterol (LDL-C), along with a notable reduction in high-density lipoprotein cholesterol (HDL-C). The administration of *R. japonica* extract at varying doses significantly lowered FFA, TC, TG, and LDL-C levels and markedly increased HDL-C, suggesting that the extract regulates glucose levels by modulating lipid metabolism. This lipid modulation likely reflects both a primary mechanism, where active constituents such as resveratrol and polydatin directly influence lipid metabolism by enhancing fatty acid oxidation and cholesterol efflux,^32^^,^^35^ and a secondary effect resulting from improved glycemic control, which reduces insulin resistance and associated dyslipidemia. These dual mechanisms underscore *R. japonica*’s potential as an antidiabetic agent through integrated glucose and lipid regulation.

In another study, Ma's study^33^ showed that treatment with *R. japonica* polysaccharides significantly improved glucose tolerance in diabetic rats (*P* < 0.05), with no significant difference compared to metformin-treated animals (*P* > 0.05). These findings indicate that polysaccharides from *R. japonica* may have glucose-regulating effects comparable to standard pharmacotherapy (Table 2).

The secondary metabolites of *R. japonica*, particularly polydatin and its derivatives, exhibit strong potential for regulating blood glucose and improving lipid profiles. These findings support the plant’s therapeutic value in the management of diabetes and its ongoing role in the development of novel antidiabetic drugs. The low oral bioavailability of compounds like polydatin and resveratrol may limit their efficacy, requiring clinical studies to optimize dosing and delivery methods.

### 3.3. Hepatoprotective effect

The hepatoprotective properties of *R. japonica* have been demonstrated primarily through two mechanisms. First, Liu Yinhua^34^ reported that an aqueous decoction of *R. japonica* significantly alleviated liver injury induced by carbon tetrachloride (CCl_4_) in experimental models. The treatment, administered over a short-term period following CCl4 induction, led to notable reductions in the serum alanine aminotransferase (ALT) and aspartate aminotransferase (AST) levels, both of which are markers of liver cell damage. Concurrently, it increases the activity of key antioxidant enzymes, including superoxide dismutase (SOD) and glutathione peroxidase (GSH-Px), which mitigate oxidative stress by neutralizing reactive oxygen species. Malondialdehyde (MDA) levels were also reduced, indicating a decrease in lipid peroxidation. Together, these effects highlight the decoction’s ability to modulate liver enzyme activity and enhance endogenous antioxidant defense, thereby conferring protection against hepatocellular injury.

Second, *R. japonica* appears to exert hepatoprotective effects by improving lipid metabolism. In a rat model of atherosclerosis, Liu Junhua et al.^34^ found that medium and high doses of polydatin significantly reduced the serum levels of TC, TG, and low-density lipoprotein cholesterol (LDL-C), while increasing high-density lipoprotein cholesterol (HDL-C) levels. Histological examination, utilizing Oil Red O staining revealed that polydatin treatment reduced hepatic cholesterol accumulation, suppressed lipid droplet formation, and prevented diffuse fatty infiltration of the liver. These findings suggest that polydatin not only regulates systemic lipid profiles but also mitigates hepatic steatosis, contributing to improved liver function and structural integrity (Table 2). The low bioavailability of polydatin and resveratrol, due to rapid metabolism, necessitates further clinical investigation.

### 3.4 Gut microbial metabolism of polyphenols

The secondary metabolites of *R. japonica*, particularly polyphenols such as resveratrol and polydatin, undergo significant metabolism by the gut microbiota, which influences their bioavailability and pharmacological effects. Resveratrol is transformed by gut microbes into bioactive metabolites such as dihydroresveratrol and piceatannol, which exhibit enhanced anti-inflammatory and antioxidant properties. These metabolites modulate the gut microbial composition, promoting the production of short-chain fatty acids that further contribute to anti-inflammatory effects by inhibiting pro-inflammatory cytokines such as IL-6 and TNF-*α*.^37^ Similarly, polydatin, a glycosylated derivative of resveratrol, is hydrolyzed by microbial *β*-glucosidases to release resveratrol, increasing its bioactivity.^38^ The gut microbiota also plays a role in modulating the systemic effects of these compounds by influencing their absorption and metabolism in the colon.

Despite these insights, *R. japonica*-specific studies on gut microbial interactions remain limited. Preliminary evidence suggests that emodin and quercetin may also undergo microbial transformation, potentially affecting their bioactivity, but further research is needed to elucidate these processes. Understanding the interplay between *R. japonica*’s secondary metabolites and the gut microbiota is critical for optimizing their therapeutic applications, particularly in anti-inflammatory and metabolic disorders.

### 3.5 Strategies to enhance bioavailability

A major limitation of *R. japonica*’s secondary metabolites, such as resveratrol and polydatin, is their low oral bioavailability, which is often less than 1% in humans due to rapid metabolism and poor solubility.^39^ Recent advancements in nanotechnology offer promising solutions to overcome these challenges. Liposomal encapsulation enhances the solubility and stability of resveratrol, improving its cellular uptake and prolonging its systemic circulation in preclinical models.^40^^,^^41^ Plant-derived nanocarriers (PDNs) significantly increase bioavailability by increasing the solubility and stability of hydrophobic drugs, reducing degradation, and enabling targeted delivery to tumor sites.^42^ Additionally, polymeric nanoparticles have shown potential in enhancing the tissue-specific delivery of anthraquinones such as emodin, improving their bioavailability and therapeutic efficacy.^43^ These nanotechnology-based formulations significantly improve the clinical efficacy of the secondary metabolites of *R. japonica*. However, clinical trials are needed to validate their safety, efficacy, and scalability for pharmaceutical applications. Future research should focus on optimizing these delivery systems to maximize the therapeutic potential of *R. japonica*-derived compounds.

## 4. Mechanisms for the regulation of secondary metabolite synthesis in *R. japonica*

### 4.1. Pathway for the synthesis of anthraquinones

Anthraquinones are among the major secondary metabolites found in *R. japonica* and are widely recognized for their broad pharmacological properties, including hemostatic, antibacterial, detoxifying, laxative, and diuretic activities.^44^These compounds are extensively utilized in traditional Chinese medicine.

According to research by Gerhard Brinmann et al., the biosynthesis of anthraquinones in *R. japonica* is closely associated with the polyketide pathway.^45^ The process begins with acetyl-CoA, which reacts with malonyl-CoA, which is activated by malonyl-CoA synthetase, to form a polyketide precursor. This precursor undergoes a series of enzyme-mediated intramolecular cyclization reactions to generate the core anthraquinone structure. Subsequent modifications, including hydroxylation, methylation, and glycosylation, further diversify the compound into biologically active forms such as emodin and rhein (1,8-dihydroxy-3-carboxyanthraquinone). These reactions are catalyzed by specific enzymes, such as cytochrome P450 monooxygenases for hydroxylation, O-methyltransferases (OMTs) for methylation, and UDP-glycosyltransferases (UGTs) for glycosylation.

Another important biosynthetic route involves the shikimate pathway. This pathway begins with phosphoenolpyruvate (PEP) and erythrose-4-phosphate (E4P), which are condensed by DAHP synthetase to produce 3-deoxy-D-arabino-heptulosonate-7-phosphate (DAHP). DAHP is subsequently converted into 3-dehydroquinic acid (DHQ) via 3-dehydroquinate synthase, then to 3-dehydroshikimic acid (DHS) by dehydroquinate dehydratase. DHS is reduced to shikimic acid (SHK) through the action of shikimate dehydrogenase. SHK is phosphorylated by shikimate kinase in an ATP-dependent reaction to yield 5-phosphoshikimic acid (S3P). This compound condenses with PEP to form chorismic acid (CHA), which is then converted into para-hydroxybenzoic acid. The latter, in its CoA-activated form, participates in downstream reactions that ultimately lead to anthraquinone synthesis.^45^

**Figure 1.**
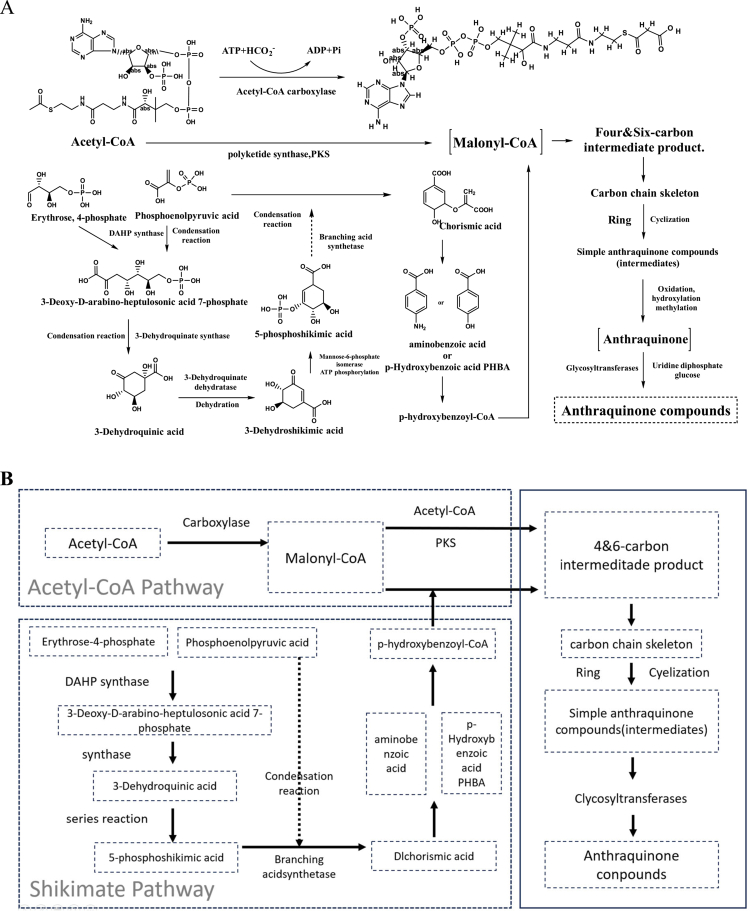
Synthesis pathway of anthraquinones.

The biosynthesis of anthraquinones requires the coordinated action of multiple enzymes. The expression levels and activity of these enzymes play a decisive role in regulating anthraquinone yield and composition in *R. japonica*^11^ (Figure 1). Notably, polyketide synthase (PKS) has been identified as a key rate-limiting enzyme in the early steps of the anthraquinone pathway, catalyzing the condensation of acetyl-CoA and malonyl-CoA to form the polyketide precursor.^45^ Additionally, cytochrome P450 monooxygenases (CYP450s), which mediate hydroxylation steps, are critical for determining the structural diversity of anthraquinones such as emodin and rhein.^45^ These enzymes represent potential targets for metabolic engineering or cultivation optimization to increase anthraquinone production.^11^^,^^45^

### 4.2. Flavonoid synthesis pathway

The biosynthesis of flavonoid compounds in *R. japonica* originates from the phenylpropanoid pathway.^46^ Phenylalanine is first converted into trans-cinnamic acid through the action of phenylalanine ammonia-lyase (PAL). This compound is then hydroxylated at the para position of the phenyl ring by cinnamate-4-hydroxylase (C4H), producing *p*-coumaric acid. Subsequently, 4-coumarate-CoA ligase (4CL) catalyzes the conjugation of *p*-coumaric acid with coenzyme A to generate *p*-coumaroyl-CoA. This intermediate, *p*-coumaroyl-CoA, undergoes a condensation reaction with three molecules of malonyl-CoA, which is catalyzed by chalcone synthase (CHS), to form chalcone. The CHS enzyme facilitates sequential Claisen-type condensations, requiring no additional cofactors beyond the substrates, as the reaction relies on the intrinsic catalytic properties of CHS.^46^ Chalcone then enters a series of downstream transformations, such as isomerization and hydroxylation, leading to the production of flavonoid compounds, including quercetin. In *R. japonica*, quercetin is considered a minor flavonoid component compared to dominant flavonoids such as myricetin and kaempferol glucuronides, as evidenced by compositional studies using UHPLC-DAD and LC–MS analyses.^4^^,^^17^

**Figure 2.**
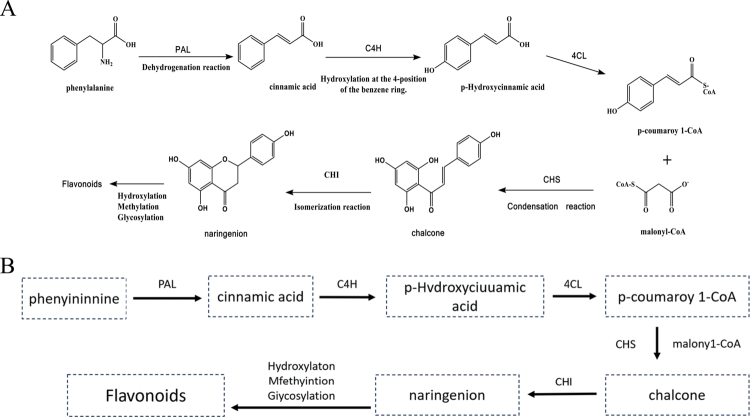
Flavonoid synthesis pathway.

The expression and regulation of key enzymes in this pathway, particularly PAL and CHS, play a central role in controlling the biosynthetic rate and yield of flavonoids. These enzymes ultimately determine the type and concentration of flavonoid metabolites produced (Figure 2).

The synthesis of phenylpropanoid compounds, including resveratrol, also begins with the phenylpropanoid pathway,^46^ sharing its initial steps with flavonoid biosynthesis. Like flavonoids, phenylalanine is first converted into trans-cinnamic acid via PAL, which is then hydroxylated by C4H to form *p*-coumaric acid. 4CL catalyzes the formation of *p*-coumaroyl-CoA from *p*-coumaric acid and CoA. At this point, the pathway diverges from flavonoid biosynthesis: while flavonoid production proceeds via chalcone synthase (CHS), which catalyzes the condensation of *p*-coumaroyl-CoA with three molecules of malonyl-CoA to form chalcone, resveratrol biosynthesis is catalyzed by resveratrol synthase (RS), a stilbene synthase (STS) with distinct substrate specificity. RS facilitates a similar condensation reaction but produces a linear stilbene precursor through a unique cyclization mechanism, differing from the chalcone structure in flavonoids.^46^ This intermediate then undergoes condensation with three molecules of malonyl-CoA, which is catalyzed by resveratrol synthase (RS), resulting in a linear precursor. The precursor undergoes cyclization, dehydration, and a series of enzymatic modifications, including transferase and hydroxylase activities, ultimately forming the final phenylpropanoid product, resveratrol.

**Figure 3.**
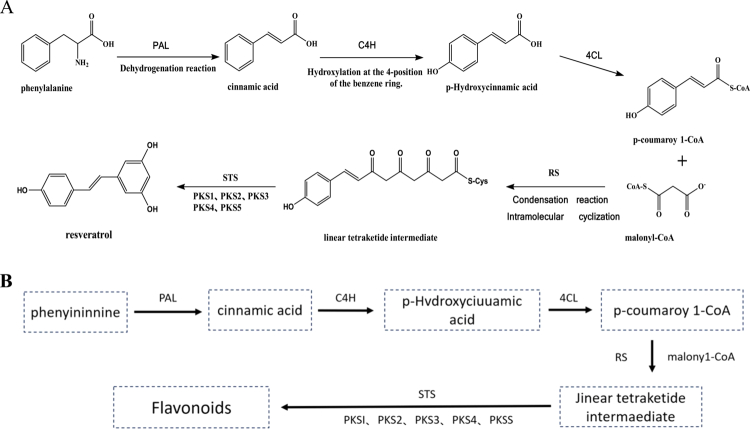
Synthesis pathway of phenylpropanoids.

Throughout this biosynthetic process, the activity and regulation of enzymes such as PAL, C4H, and RS are critical in determining the efficiency and output of resveratrol synthesis (Figure 3).

### 4.3. Glycoside analogue synthesis pathway

#### 4.3.1. Glycoside biosynthesis

The biosynthesis of glycosides involves two primary components: the formation of glycosyl donors and glycosyl acceptors.

Glycosyltransferases catalyze the transfer of a glycosyl group from a donor to an acceptor molecule via a glycosidic bond, after which the product undergoes various modification reactions to yield the final glycosidic metabolites. According to research by Song Chuankui’s group, the primary glycosyl donors are nucleoside diphosphates, such as UDP-glucose, which are synthesized from monosaccharides such as glucose through a series of enzyme-catalyzed reactions. The key enzymes in this pathway include glucokinase, which phosphorylates glucose to glucose-6-phosphate, and UDP-glucose pyrophosphorylase (UGPase), which catalyzes the formation of UDP-glucose from glucose-1-phosphate and UTP. The glycosyl acceptors can include phenolic compounds, flavonoids, terpenoids, alkaloids, and other molecules.^47^

Research by Ye Min and Qiao Xue's team has shown that different glycosyltransferases exhibit distinct substrate specificities, allowing them to recognize and selectively bind various glycosyl donors and acceptors to produce structurally diverse glycosides, such as piceid and emodin glycosides, in *R. japonica*, which increase their solubility and bioactivity, contributing to the plant’s pharmacological properties.^48^ Once formed, glycosides may undergo further modifications, such as methylation, acetylation, or sulfation, which alter their physicochemical properties and biological functions.

#### 4.3.2. Transcriptional regulation of glycoside biosynthesis

**Figure 4.**
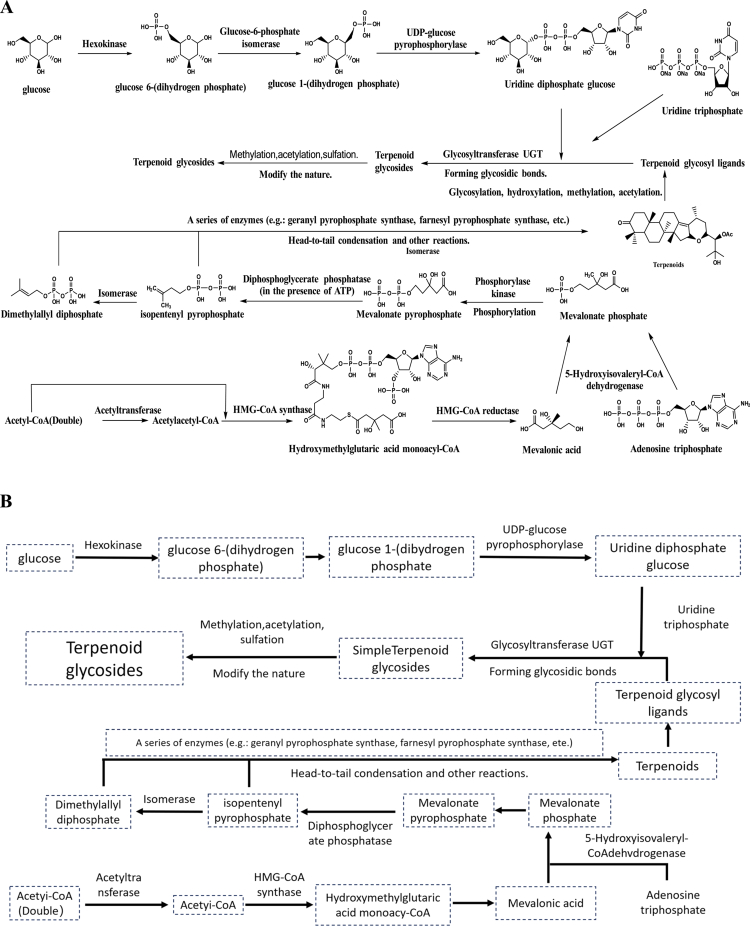
Simple synthesis pathway of glycosides.

The production of glycosides in *R. japonica* is tightly regulated by transcription factors that modulate glycosyltransferase gene expression in response to hormonal and environmental signals. The transition from glycoside biosynthesis to its regulation involves the coordinated activity of MYB and bHLH transcription factors, which bind to promoter regions of genes encoding enzymes such as UDP-glycosyltransferases (UGTs).^48^ The expression of glycoside biosynthetic genes and the activity of the associated enzymes are also modulated by plant hormones, including auxins and cytokinins, which exert their regulatory effects through signal transduction pathways (Figure 4). While this regulation is well-documented in general plant systems, such as Arabidopsis and grapevine, where auxins and cytokinins upregulate glycosyltransferase genes and stilbene glycoside production,^47^^,^^48^ *R. japonica*-specific experimental evidence is limited. Studies^49^ have suggested that hormones, including auxins, influence resveratrol glycoside accumulation in *R. japonica* tissue cultures, but further species-specific research is needed to confirm these effects. This inference underscores the potential role of hormone signaling in enhancing glycoside biosynthesis for therapeutic applications in *R. japonica*.

In addition, the secondary metabolism of *R. japonica* is regulated by transcription factors such as MYB and bHLH, as well as by reactive oxygen species (ROS) signaling and calcium ion (Ca²^+^) signaling. The specific MYB subtypes characterized in *R. japonica* include PcMYB62, an R2R3-MYB repressor that inhibits resveratrol biosynthesis by binding to the stilbene synthase (PcSTS) promoter, and PcMYB1, PcMYB10, PcMYBR01, PcMYB114, and PcMYB306, which regulate polydatin biosynthesis by modulating the glycosyltransferase PcUGT95D10 promoter activity.^44^ For bHLH transcription factors, *R. japonica*-specific subtypes are less characterized, but their roles are inferred from Arabidopsis homologs, such as TT8 and GL3 (subgroup IIIf), which are known to form MYB-bHLH-WD40 (MBW) complexes that regulate the flavonoid and stilbene pathways.^50^^,^^51^ However, further studies are needed to identify *R. japonica*-specific bHLH subtypes involved in secondary metabolism. The MYB transcription factor family plays a crucial role in regulating secondary metabolism. These transcription factors bind specifically to the promoter regions of biosynthetic genes involved in flavonoid and phenylpropanoid production, such as CHS, thereby promoting gene transcription and enhancing the synthesis of flavonoid compounds. Some MYB transcription factors also interact with other transcription factors or signaling pathways to indirectly influence the expression of genes involved in anthraquinone biosynthesis, coordinating the activity of various metabolic branches.^51^

The bHLH transcription factors frequently cooperate with MYB proteins to form regulatory complexes that increase the expression of target genes within specific secondary metabolic pathways. In *R. japonica*, bHLH factors are known to fine-tune flavonoid biosynthesis by regulating key enzymes in collaboration with MYB partners. Furthermore, bHLH transcription factors are also involved in the regulation of anthraquinone and phenylpropanoid biosynthesis, contributing to the coordinated and balanced production of multiple classes of secondary metabolites.^51^^,^^52^

## 5. Factors affecting the content of secondary metabolites

The accumulation of *R. japonica*’s secondary metabolites is governed by a complex interplay of internal factors, such as plant age, organ type, and external factors like soil nutrients and water availability. These factors interact dynamically, with plant age modulating hormone signaling that amplifies biosynthetic enzyme activity, while environmental conditions, including light and soil composition, fine-tune tissue-specific metabolite profiles. This section includes these influences, highlighting their mechanistic interactions and relative contributions to pharmacological potency.

### 5.1. Growth period

*R. japonica* is widely used in traditional medicine, and the accumulation of its secondary metabolites varies significantly with plant age.^53^ Studies have shown^54^ that the levels of jasmonic acid (JA) and abscisic acid (ABA) in *R. japonica* increase as the plant matures,^55^ a general trend observed across many plant species where these hormones regulate stress responses and secondary metabolism. In *R. japonica*, this hormonal increase is particularly significant, as it enhances the biosynthesis of secondary metabolites such as resveratrol, polydatin, and emodin, driven by JA- and ABA-mediated activation of MYB and bHLH transcription factors.^46^^,^^56^ This hormonal increase corresponds to the coordinated activity of the MYB and bHLH transcription factors within plant cells,^46^ which increases the biosynthetic capacity of the flavonoid and anthraquinone pathways. As a result, the content of anthraquinone compounds, such as emodin, increases with plant age. Thus, growth years indirectly influences the production of secondary metabolites by regulating the levels of key plant hormones,^57^ which is consistent with the findings of Chen Jing et al.^58^^,^^59^ Notably, stilbenes such as resveratrol and polydatin exhibit a more pronounced age-related increase compared to anthraquinones like emodin, with resveratrol glycosides peaking in older plants because of stronger JA-mediated regulation and MYB activation.^56^ In contrast, emodin accumulation increases steadily but less dramatically with age.^54^ Thus, growth years indirectly influences secondary metabolite production by regulating key plant hormones,^57^ which is consistent with findings by Chen Jing et al.^58^^,^^59^ These metabolite-specific trends highlight the pharmacological potential of older *R. japonica* plants, particularly for resveratrol and polydatin-rich extracts used in antidiabetic and anti-inflammatory applications.

Flavonoid accumulation also increases with age, largely due to increased levels of cinnamic acid generated through the PAL pathway. Cinnamic acid serves as a key precursor in flavonoid biosynthesis and is converted to *p*-coumaroyl-CoA by 4-coumarate: CoA ligase (4CL), which is then utilized by chalcone synthase (CHS) to produce chalcones, the initial precursors of flavonoids.^17^^,^^46^ This increases CHS enzyme expression, leading to increased flavonoid biosynthesis,^17^ as reported by Wang Qin et al. ^60^ in studies on *R. japonica*. Moreover, the contents of major compounds such as resveratrol and polydatin have been shown to vary with growth years, as noted by Cao Liang,^56^ Chen Jing,^58^ and others. These two compounds are primarily synthesized via the PAL pathway, and their production is significantly upregulated by JA, which induces PAL gene expression^61^ and accelerates phenylpropanoid metabolism.

As JA content increases with plant age, it increases PAL activity and activates enzymes such as stilbene synthase,^11^ ultimately enhancing the synthesis of resveratrol. These findings explain why the purity and yield of key compounds such as resveratrol and polydatin are greater in older plants, a conclusion supported by studies from Wen Tao^3^ and Cao Liang.^56^

Growth years are a critical factor influencing the accumulation of secondary metabolites in *R. japonica*. This influence is primarily exerted through the modulation of plant hormone activity, such as jasmonic acid selection, and the regulation of core biosynthetic mechanisms such as the PAL pathway and MYB transcription factors.^62^ The plant’s age not only reflects its developmental status but also plays a key regulatory role in determining its medicinal potency by driving the accumulation of bioactive compounds. This dynamic regulation underscores why older plants are preferred in TCM for their relatively high bioactive compound content, providing a mechanistic basis for cultivation strategies targeting mature *R. japonica*.

### 5.2. Different parts

The biosynthesis of secondary metabolites in different parts of *R. japonica* is influenced by a range of internal and external factors, including environmental conditions and light intensity. Owing to the morphological and functional variations among plant organs, the biosynthetic pathways and accumulation mechanisms of secondary metabolites can differ significantly between tissues. This tissue-specific variation is driven by differential gene expression and environmental exposure, with stems and leaves responding to light-induced stress, while roots prioritize defense-related metabolites like resveratrol.

Flavonoids, one of the major classes of secondary metabolites in *R. japonica*, play important roles as antioxidants and UV-protective agents, helping the plant defend against external stressors.^61^ Resveratrol, which possesses antioxidant, pest- and disease-resistance, and UV-protective properties, as confirmed by in planta studies, is typically found at relatively high concentrations in the rhizomes, where it contributes to the plant’s environmental stress response.^56^ Research has shown that stems, which are more directly exposed to sunlight and UV radiation,^63^ exhibit increased activity of PAL, a key enzyme in the phenylpropanoid biosynthetic pathway.^64^ This leads to increased resveratrol biosynthesis in the stem, with concentrations of resveratrol and polydatin notably higher in stems than in leaves, as also reported by Zhou Kun et al.^49^^,^^65^ JA enhances resveratrol synthesis in stems through UV-B-induced signaling, as described in Section 5.1, explaining their higher stilbene content for TCM extractions.

Flavonoids increase a plant’s resistance to environmental stress and pathogen invasion by mitigating oxidative damage, regulating the expression of defense-related genes through jasmonate signaling and reactive oxygen species (ROS) modulation, and increasing tolerance to adverse conditions such as UV radiation.^66^^,^^67^ These protective effects are mediated through molecular mechanisms, including the scavenging of ROS to reduce oxidative stress and the activation of the jasmonic acid (JA) and salicylic acid (SA) signaling pathways, which upregulate defense genes such as pathogenesis-related (PR) genes.^46^^,^^61^ Higher soil moisture, typically ranging from 50% to 80% of field capacity, enhances the activity of key enzymes in the flavonoid biosynthetic pathway, such as PAL, CHS, and CHI, thereby increasing flavonoid accumulation.^68^^,^^69^ In many medicinal plants, flavonoid concentrations are typically higher in leaves than in roots.^46^^,^^70^ Leaves, which are more exposed to ultraviolet B (UVB) radiation, activate the synthesis of large amounts of flavonoid compounds through the upregulation of enzymes such as PAL, cinnamate-4-hydroxylase (C4H), and flavanone 3-hydroxylase (F3H).^71^ Similarly, Wang Huatian et al. ^72^ demonstrated that the flavonoid content in leaves increases with light exposure and decreases under shading conditions. This light-dependent flavonoid accumulation in leaves is likely driven by UV-B-induced upregulation of MYB transcription factors, which prioritize flavonoid synthesis over stilbenes in photosynthetic tissues, explaining the organ-specific metabolite profiles.

These observations suggest that variations in flavonoid content are closely linked to the differential environmental exposure of plant organs. This conclusion is consistent with the findings of Xiao Linxia,^73^ who studied the effects of light on *R. japonica*. Additionally, genes such as gentianol-10-hydroxylase (G1OH), putative squalene epoxidase (PtSQE), and putative *β*-amylase synthase (Ptβ-AS) regulate the biosynthetic activity of flavonoid-related enzymes and exhibit higher expression in roots, promoting flavonoid accumulation and contributing to the plant’s protective capacity.^74^^,^^75^ Furthermore, spatial differences in gene expression also modulate plant hormone activity, which in turn influences the accumulation of secondary metabolites in different organs.^76^ The differential expression of biosynthetic genes across organs underscores why roots are prioritized in TCM for their high stilbene content, while leaves may be targeted for flavonoid-rich extracts.

Overall, the accumulation of secondary metabolites in *R. japonica* is closely tied to a specific plant organ. The morphological and functional characteristics of each part, along with environmental exposure, shape the tissue-specific metabolic processes and determine the biosynthetic dynamics of secondary compounds.

### 5.3. Soil nutrients

Soil, as the foundation for plant growth, plays a critical role not only in the development of *R. japonica* but also in the biosynthesis of its secondary metabolites. Mineral elements in the soil, particularly nitrogen and phosphorus, are essential for normal plant physiological processes and metabolic regulation. Numerous studies^77^^,^^78^ have demonstrated that high levels of nitrogen and phosphorus significantly increase the synthesis and accumulation of secondary metabolites in medicinal plants.

Specifically, the availability of nitrogen and phosphorus has been shown to influence the accumulation of key metabolites such as resveratrol and emodin. Plants cultivated in nitrogen- and phosphorus-rich soils tend to exhibit elevated levels of these compounds, likely due to increased bioavailability and absorption of these nutrients. This effect is particularly evident in the roots of *R. japonica* where increased soil phosphorus has been correlated with increased polydatin and emodin synthesis. Studies suggest that this increase results from both the transcriptional upregulation of biosynthetic genes (e.g., those encoding polyketide synthases for emodin) and the increased activity of key enzymes (e.g., chalcone synthase for polydatin precursors), which are influenced by phosphorus availability.^78^^,^^79^ These findings, supported by Yu ^78^ and others, further confirmed that anthraquinone biosynthesis is sensitive to soil nutrient composition, with nitrogen and phosphorus acting as regulatory factors that influence the expression of relevant biosynthetic pathways. In general, these findings underscore the importance of soil nutrient availability in promoting the biosynthetic capacity of *R. japonica*.

Water availability is another key environmental factor that impacts both plant growth and the production of secondary metabolites. As an essential component of plant physiological and biochemical processes, water directly influences metabolic activity, enzyme function, and hormone synthesis. Research has shown that relatively high soil moisture, typically ranging from 50% to 80% of field capacity, enhances the activity of key enzymes involved in the flavonoid biosynthetic pathway, such as PAL, CHS, and chalcone isomerase (CHI), thereby increasing flavonoid accumulation. These moisture levels optimize metabolic activity and enzyme function, increasing yields of bioactive compounds such as quercetin in *R. japonica*. These results are consistent with studies showing that water stress and water availability modulate PAL and CHS activity in medicinal plants.

Additionally, water availability influences the synthesis of plant hormones such as ABA and JA, which are known to activate secondary metabolic pathways. Elevated moisture levels have been linked to increased flavonoid, alkaloid, and other secondary metabolite synthesis, as demonstrated in studies by Wen and others. Collectively, these findings suggest that the soil water content plays a significant regulatory role in the accumulation of secondary metabolites in *R. japonica* through its effects on enzyme activity, hormone signaling, and plant physiological responses. These nutrient and water effects highlight the need for integrated soil management strategies to optimize the medicinal potential of *R. japonica* in TCM applications.

Both soil mineral composition, particularly nitrogen and phosphorus, and water availability are critical environmental factors that influence the biosynthesis and accumulation of secondary metabolites in *R. japonica*. Ensuring optimal soil nutrient content and moisture conditions can therefore increase the yield and quality of bioactive compounds in this important medicinal plant. To further optimize metabolite production, future research should explore the role of the soil microbiota and rhizosphere interactions, which are increasingly recognized as key modulators of secondary metabolite biosynthesis.^80^ For example, the integration of metagenomic studies or microbial elicitors could reveal how beneficial microbes increase the yield and diversity of bioactive compounds such as resveratrol and anthraquinones, complementing nutrient and water management strategies. These results are consistent with studies showing that environmental factors modulate enzyme activity in medicinal plants.

### 5.4. Interactions among influencing factors

In summary, the synergistic effects of plant age, organ type, and environmental conditions significantly increase secondary metabolite production in *R. japonica*. For instance, older plants in phosphorus-rich soils presented increased resveratrol and polydatin yields due to JA-mediated upregulation of stilbene synthase (RS) and phosphorus-dependent activation of polyketide synthase (PKS). This synergy arises because JA signaling, which is amplified with age, enhances RS expression, while phosphorus provides the energetic resources such as ATP needed for biosynthetic reactions, particularly in roots. Similarly, high soil moisture (50%–80% field capacity) amplifies flavonoid accumulation in leaves by increasing PAL and CHS activity, particularly under UV-B exposure. This interaction is driven by the role of water in facilitating nutrient uptake and stabilizing enzyme function, which is further potentiated by light-induced MYB transcription factor activation. These interactions suggest that integrated cultivation strategies, combining optimal nutrient management with controlled stress priming conditions, such as UV-B and JA elicitors, could maximize the therapeutic potential of *R. japonica*. For instance, combining phosphorus supplementation with methyl jasmonate treatment could synergistically increase resveratrol yields in older plants, as supported by comparative studies in other species.^81^ Future research should quantify these interactions using factorial experimental designs to optimize cultivation practices for TCM applications.

## 6. Conclusions and outlook

The secondary metabolites of *R. japonica*, including flavonoids, anthraquinones, and stilbenes, exhibit a broad spectrum of biological activities, including anti-inflammatory, antioxidant, antitumor, and immunomodulatory effects. The formation and accumulation of secondary metabolites in *R. japonica* are governed by a complex interplay of factors such as plant age, specific tissues or organs, and environmental conditions. These variables influence not only the concentration but also the chemical structure of the metabolites, ultimately affecting the plant's pharmacological efficacy.^4^ Recent studies, such as that of Liu et al.,^82^ highlight the FMH potential of phenolic compounds such as ferulic acid in combating colon cancer through antioxidant and anti-inflammatory mechanisms, paralleling the bioactivities of the resveratrol and polydatin of *R. japonica*. Similarly, Ye et al. ^83^ reported the neuroprotective potential of flavonoids and phenolic acids from Leguminosae species, suggesting that the metabolites of *R.japonica* could be explored for their ability to treat neurodegenerative disorders, expanding its TCM applications into modern integrative medicine.

To further unlock the medicinal and industrial potential of *R. japonica*, continued research is required to elucidate the pharmacological mechanisms of its secondary metabolites, clarify how various internal and external factors regulate their biosynthesis, and optimize cultivation strategies aimed at enhancing both yield and bioactive compound content. Specific techniques, such as elicitor application (e.g., methyl jasmonate to stimulate phenylpropanoid pathways,^63^ controlled light exposure, such as UV-B radiation, to increase flavonoid accumulation,^67^^,^^71^ and nutrient regulation, such as optimized nitrogen and phosphorus levels to increase anthraquinone synthesis,^78^ have shown promise in other medicinal plants and could be explored for *R. japonica*. Future studies should focus on elucidating pharmacological mechanisms, optimizing cultivation, exploring the soil microbiota, and conducting clinical trials to validate preclinical findings and overcome bioavailability limitations. While biosynthetic pathways for stilbenes and glycosides have been studied in model systems such as Arabidopsis and grapevine, species-specific data for *R. japonica* remain limited. To address this gap, we recommend conducting transcriptomics and proteomics studies to characterize the regulatory genes and enzymes involved in stilbene and glycoside biosynthesis in *R. japonica*. These studies, combined with metabolic engineering, could increase metabolite yield and specificity. In summary, advancing these areas will provide a robust scientific foundation for the clinical application and commercial utilization of this plant.

While *R. japonica* exhibits significant therapeutic potential, its safety profile requires careful consideration. High doses of anthraquinones, such as emodin, have been associated with potential hepatotoxicity and nephrotoxicity in animal models, with studies reporting elevated liver enzymes and renal tubular damage.^84^ The chronic use of *R. japonica* extracts, particularly those rich in anthraquinones, may lead to gastrointestinal disturbances or laxative dependence, as noted in traditional medicine literature.^85^ The low oral bioavailability of resveratrol and polydatin further complicates their therapeutic application, necessitating careful dose optimization. Long-term clinical studies are needed to establish safe therapeutic thresholds and evaluate the risks of prolonged use.

To maximize the therapeutic efficacy of *R. japonica*, innovative strategies to increase secondary metabolite production are essential. Stress priming with elicitors, such as methyl jasmonate, can upregulate the biosynthesis of key compounds such as resveratrol by inducing stilbene synthase expression through the jasmonic acid and ROS signaling pathways.^86^ Similarly, controlled UV-B radiation exposure has been shown to increase flavonoid accumulation by upregulating enzymes such as PAL and CHS. RNA-based technologies, including small interfering RNAs (siRNAs) and microRNAs (miRNAs), offer promising tools to increase the production of flavonoids, stilbenes, and anthraquinones.^87^^,^^88^ Integrating stress priming with RNA signaling and genetic engineering can optimize phytochemical production, increasing the yield of bioactive compounds for therapeutic applications. For instance, targeted overexpression of MYB transcription factors could increase flavonoid and stilbene biosynthesis, as demonstrated in other medicinal plants. These approaches, combined with optimized cultivation conditions, hold significant potential for improving the therapeutic and industrial value of *R. japonica*. Further research is needed to validate these strategies in *R. japonica*-specific systems and ensure their sustainability for large-scale production.

## Data Availability

The datasets used and/or analyzed during the current study are included in the manuscript.

## References

[1] Wang F, Li M, Liu Z, He Q, Xing L, Xiao Y, Du C, Zhang H, Zhou Y. The mixed auto-/allooctoploid genome of Japanese knotweed (*Reynoutria japonica*) provides insights into its polyploid origin and invasiveness. Plant J. 2025;121(4), e70005. doi: 10.1111/tpj.70005. 39993002

[2] Jiang L, Zhang J, Zhu B, Bao X, Tian J, Li Y, Zhang G, Wang L, Zhang W, Tang Y, et al. The aqueous extract of *Reynoutria japonica* ameliorates damp-heat ulcerative colitis in mice by modulating gut microbiota and metabolism. J Ethnopharmacol. 2025;338(Pt 1), 119042. doi: 10.1016/j.jep.2024.119042. 39515678

[3] Wen T, Study on the regulation system of resveratrol secondary metabolism in *Reynoutria japonica* callus. 2009.

[4] Liu F, Li F, Feng Z, Yang Y, Jiang J, Zhang P. Neuroprotective naphthalene and flavan derivatives from *Polygonum cuspidatum*. Phytochemistry. 2015;110:150–159. doi: 10.1016/j.phytochem.2014.12.007. 25553583

[5] Hao D, Ma P, Mu J, Chen S, Xiao P, Peng Y, Huo L, Xu L, Sun C. De novo characterization of the root transcriptome of a traditional Chinese medicinal plant *Polygonum cuspidatum*. Sci China Life Sci. 2012;55(5):452–466. doi: 10.1007/s11427-012-4319-6. 22645089

[6] Ma TH, Sheng T, Tian CM, Xing MY, Yan LJ, Xia DZ. Effect of ethanolic extract of Polygonum cuspidatum on acute gouty arthritis in mice through NLRP3/ASC/caspase-1 axis. Zhongguo Zhong Yao Za Zhi. 2018;2019l44(3):546–552. doi: 10.19540/j.cnki.cjcmm.20180925.001. 30989921

[7] Liu Y, et al. Protective effects of *Rabdosia serra*–*Reynoutria japonica* decoction on experimental liver injury in rats. Shizhen Tradition Chin Med Chinese Herbal Med. 2008;19(2):334–335.

[8] Kong X, Zhou L. Advances in research on *Reynoutria japonica* (Huzhang) in traditional Chinese medicine. Herald of Med / Guiding J Tradition Chin Med Pharm. 2009;15(5):107–110.

[9] Shi Y, Zhang L, Yao D, Liu H, Qi Z, Liu J. Inhibitory effects of polydatin and its derivatives on SGLT2 for hypoglycemic activity. Chin J Mod Appl Pharm. 2018;35(11):1684–1688.

[10] Ma P. Peking Union Medical College Pharmacognostic studies of *Reynoutria japonica*. 2013. 137. doi: 10.7666/d.Y2340470.

[11] Hou Q, et al. Research progress of *Reynoutria japonica* and prediction of quality markers. Mod Chin Med. 2024;26(5):912–926.

[12] Zhang Y, Huang X, Chen Y, Li J, Yu K. Progress in the study of the main chemical components and their biosynthesis mechanism of *Reynoutria japonica*. Chin J Tradition Chin Med. 2020;45(18):4364–4372.

[13] Feng L, Research on the chemical composition and quality of *Polygonum cuspidatum.* 2003.

[14] Jin X, Jin G. Research on the chemical composition of *Reynoutria japonica*. Chin Herbal Med. 2007;38(10):1446–1448.

[15] Nonomura S, Kanagawa H, Makimoto A. Chemical constituents of polygonaceous plants. I. Studies on the components of KO-J O-Kon. (*Polygonum cuspidatum* Sieb. Et Zucc. Yakugaku Zasshi. 1963;83(10):988–990. doi: 10.1248/yakushi1947.83.10_988. 14089847

[16] Li P, Zhu X, Xiao M, Su Y, Yu S, Tang J, Xue H, Cai X. Rapid isolation and hypoglycemic activity of secondary metabolites of *Eurotium cristatum* by high-speed countercurrent chromatography. J Chromatogr Sci. 2023;61(6):539–545. doi: 10.1093/chromsci/bmac020. 35325046

[17] Wang MA, Wang MK, Peng S. Research on the chemical composition of *Reynoutria japonica*. Nat Prod Res Dev. 2001;13(6):16–18.

[18] Zhang B, Xu Y, Lv H, Pang W, Wang J, Ma H, Wang S, et al. Intestinal pharmacokinetics of resveratrol and regulatory effects of resveratrol metabolites on gut barrier and gut microbiota. Food Chem. 2021;357:129532. doi: 10.1016/j.foodchem.2021.129532. 33878586

[19] Li D. Research progress on the application of resveratrol glycosides in animal medicine. Mod J Anim Husband Veterin Med. 2022. (10):86–88.

[20] Ilhan M, Ali Z, Khan IA, Taştan H, Küpeli Akkol E. Bioactivity-guided isolation of flavonoids from *Urtica dioica* L. and their effect on endometriosis rat model. J Ethnopharmacol. 2019;243 112100. 31325603 10.1016/j.jep.2019.112100

[21] Balkrishna A, Solleti SK, Singh H, Verma S, Sharma N, Nain P, Varshney A, et al. Herbal decoction Divya-Swasari-Kwath attenuates airway inflammation and remodeling through Nrf-2 mediated antioxidant lung defence in mouse model of allergic asthma. Phytomedicine. 2020;78: 153295. doi: 10.1016/j.phymed.2020.153295. 32795904

[22] Li TX, Liang JX, Liu LL, Shi FC, Jia XW, Li MH, Xu CP, et al. Novel kojic acid derivatives with anti-inflammatory effects from *Aspergillus versicolor*. Fitoterapia. 2021;154:105027. doi: 10.1016/j.fitote.2021.105027. 34492330

[23] Yuan L, Zhao H, Su X. Purification and anti-tumor activity study of small molecule peptide-tumor suppressant 19 peptide. Adv Mod Biomed. 2007;7(5):674–676.

[24] Wang X, Sun S, Duan Z, Yang C, Chu C, Liu B, Ding W, Li W. Protective effect of ethyl pyruvate on gut barrier function through regulations of ROS-related NETs formation during sepsis. Mol Immunol. 2021;132:108–116. doi: 10.1016/j.molimm.2021.01.012. 33581408

[25] Learmonth DA. A concise synthesis of the 3-O-beta-D- and 4'-O-beta-D-glucuronide conjugates of trans-resveratrol. Bioconjug Chem. 2003;14(1):262–267. doi: 10.1021/bc020048x. 12526717

[26] Kong D., Ren C., Ning C., Cheng Y., Cai H., Xing H., Zhang Y., Li N., Lu Y., Chen X., et al. Pulmonary administration of resveratrol/hydroxypropyl-β-cyclodextrin inclusion complex: in vivo disposition and in vitro metabolic study. J Drug Deliv Sci Technol. 2020;60 101995. 10.1016/j.jddst.2020.101995.

[27] Tang J, Diao P, Shu X, Li L, Xiong L, et al. Quercetin and quercitrin attenuates the inflammatory response and oxidative stress in LPS-induced RAW264.7 cells: in vitro assessment and a theoretical model. Biomed Res Int. 2019;2019:7039802. doi: 10.1155/2019/7039802. 31781635 PMC6855062

[28] Richette P, Bardin T. Gout. Lancet. 2010;375(9711):318–328. doi: 10.1016/S0140-6736(09)60883-7. 19692116

[29] Fan W, Chen S, Wu X, Zhu J, Li J. Resveratrol relieves gouty arthritis by promoting mitophagy to inhibit activation of NLRP3 inflammasomes. J Inflamm Res. 2021;14:3523–3536. doi: 10.2147/JIR.S320912. 34335041 PMC8318089

[30] Xu W, Research on the role and mechanism of polydatin in preventing and treating hyperuricemia and gouty arthritis. 2023.

[31] Cheng J. The effect of *Reynoutria japonica* extract on the joint dysfunction index and inflammation index of rats with acute gouty arthritis. Biochem Eng. 2023;9(4):94–97.

[32] Zhao H, Wang Y, Liu X, Zhang C, Jiao L, Di L. The effect of *Reynoutria japonica* extract on blood glucose and blood lipids in type 2 diabetic rats. Tradition Chin Med Mater. 2016;39(7):1647–1650.

[33] Ma N, Zhou X, Zhang J, Qi N. Study on the hypoglycemic effect of *Reynoutria japonica* polysaccharides on streptozotocin-induced diabetes model. Strait Pharmaceut J. 2023;35(7):13–17.

[34] Liu Y, Liang L, Shen J, Ai W, Hu Z. Experimental study on the choleretic effects of xihuangcao and *Reynoutria japonica* decoction. Shizhen Tradition Chin Med Chin Herbal Med. 2018;15(4):99–104.

[35] Cheng L, Zhang S, Shang F, Ning Y, Huang Z, He R, Sun J, Dong S, et al. Emodin improves glucose and lipid metabolism disorders in obese mice via activating brown adipose tissue and inducing browning of white adipose tissue. Front Endocrinol. 2021;12: 618037. doi: 10.3389/fendo.2021.618037. PMC8143048 34040579

[36] He Y, Wang H, Lin S, Chen T, Chang D, Sun Y, Wang C, Liu Y, Lu Y, Song J, et al. Advanced effect of curcumin and resveratrol on mitigating hepatic steatosis in metabolic associated fatty liver disease via the PI3K/AKT/mTOR and HIF-1/VEGF cascade. Biomed Pharmacother. 2023;165: 115279. doi: 10.1016/j.biopha.2023.115279. 37544281

[37] Man AWC, Li H, Xia N. Resveratrol and the interaction between gut microbiota and arterial remodelling. Nutrients. 2020;12(1):119. doi: 10.3390/nu12010119. 31906281 PMC7019510

[38] Bode LM, Bunzel D, Huch M, Cho G, Ruhland D, Bub A, Franz CM, Kulling SE. In vivo and in vitro metabolism of trans-resveratrol by human gut microbiota. Am J Clin Nutr. 2013;97(2):295–309. doi: 10.3945/ajcn.112.049379. 23283496

[39] Walle T, Hsieh F, DeLegge MH, Oatis JE. High absorption but very low bioavailability of oral resveratrol in humans. Drug Metab Dispos. 2004;32(12):1377–1382. doi: 10.1124/dmd.104.000885. 15333514

[40] Annaji M, Poudel I, Boddu SHS, Arnold RD, Tiwari AK, Babu RJ. Resveratrol-loaded nanomedicines for cancer applications. Cancer Rep. 2021;4(3), e1353. doi: 10.1002/cnr2.1353. PMC8222557 33655717

[41] Chimento A, De Amicis F, Sirianni R, Sinicropi MS, Puoci F, Casaburi I, Saturnino C, Pezzi V. Progress to improve oral bioavailability and beneficial effects of resveratrol. Int J Mol Sci. 2019;20(6):1381. doi: 10.3390/ijms20061381. 30893846 PMC6471659

[42] Alum EU, Nwuruku OA, Ugwu OP, Uti DE, Edwin N. Harnessing nature: plant-derived nanocarriers for targeted drug delivery in cancer therapy. Phytomed Plus. 2025;5(3), 100828. doi: 10.1016/j.phyplu.2025.100828.

[43] Uti DE, Atangwho IJ, Alum EU, Ntaobeten E, Obeten UN, Bawa I, Agada SA, Ukam CI, Egbung GE. Antioxidants in cancer therapy mitigating lipid peroxidation without compromising treatment through nanotechnology. Discov Nano. 2025;20(1):70. doi: 10.1186/s11671-025-04248-0. 40272665 PMC12021792

[44] Xia A, Zhang H, Jia A, Chai Y, Zhang G. Development of a multi-constituent quantification method for quality control of *Reynoutria japonica*. Chin Herbal Med. 2011;42(9):1761–1765.

[45] Bringmann G, Irmer A. Acetogenic anthraquinones: biosynthetic convergence and chemical evidence of enzymatic cooperation in nature. Phytochem Rev. 2008;7(3):499–511. doi: 10.1007/s11101-008-9090-8.

[46] Mao Y, Luo J, Cai Z. Biosynthesis and regulatory mechanisms of plant flavonoids: a review. Plants. 2025;14(12):1847. doi: 10.3390/plants14121847. 40573835 PMC12196789

[47] Lu M, Zhao Y, Feng Y, Tang X, Yu K, Pan Y, Wang Q, Cui J, Zhang M, Jin J, et al. 2,4-Dihydroxybenzoic acid, a novel sa derivative, controls plant immunity via UGT95B17-mediated glucosylation: a case study in camellia sinensis. Adv Sci. 2024;11(7), p. e2307051. 10.1002/advs.202307051. PMC10870048 38063804

[48] Wang HT, Chen K, Yao M, Zhang M, Ågren H, Li F, Qiao X, Ye M. Insights into the missing apiosylation step in flavonoid apiosides biosynthesis of Leguminosae plants. Nat Commun. 2023;14(1):6658. doi: 10.1038/s41467-023-42393-1. 37863881 PMC10589286

[49] Zhou K. Callus culture induction and polydatin accumulation in *Reynoutria japonica*. 2006. p. 46 Hefei/ Province, Anhui: Anhui Agricultural University.

[50] Wang Z, Mao Y, Guo Y, Gao J, Liu X, Li S, Lin YJ, Chen H, Chiang VL. MYB transcription factor161 mediates feedback regulation of secondary wall-associated NAC-Domain1 family genes for wood formation. Plant Physiol. 2020;184(3):1389–1406. doi: 10.1104/pp.20.01033. 32943464 PMC7608153

[51] Dubos C, et al. *M*YB transcription factors in *Arabidopsis*. Trends Plant Sci. 2010;15(10):573–581. 20674465 10.1016/j.tplants.2010.06.005

[52] Zhang S, Chen Y, He X, Du J, Ma Y, Hu X, Wan X. Identification of myb transcription factors regulating theanine biosynthesis in tea plant using omics-based gene coexpression analysis. J Agric Food Chem. 2020;68(3):918–926. doi: 10.1021/acs.jafc.9b06730. 31899636

[53] Gu X, Zhao Y, Zhang J, Wang Q, Zheng Y, Fang H. Dynamics of secondary metabolite accumulation in atractylodes chinensisin response to soil mineral elements across growth years. Shandong Agric Sci. 2023;55(6):95–100.

[54] Cao W, Tao Y. Interannual variation of five anthraquinone derivatives in *Rheum tanguticum* (Tangut rhubarb). Mod Appl Pharm China. 2008;25(5):404–406.

[55] Xing W, Jin X. Recent advances in MYB transcription factors regulating biosynthesis of bioactive flavonoids in plants. Mol Plant Breed. 2015;13(3):689–696.

[56] Cao L, J Zhou, L Zhang. Growth-year-dependent variations in resveratrol glycoside and aglycone contents in *Reynoutria japonica*. Chin Patent Med. 2009;31(6):897–900.

[57] Liu W, Zheng T, ang Y Y, Li P, Qiu L, i L L, Wang J, Cheng T, Zhang Q, et al. Meta-analysis of the effect of overexpression of myb transcription factors on the regulatory mechanisms of anthocyanin biosynthesis. Front Plant Sci. 2021;12: 781343. doi: 10.3389/fpls.2021.781343. 34975967 PMC8714666

[58] Chen J, Zhao Y, Li C, Hao X. Effects of four growth years on four bioactive components in *Reynoutria japonica*. J Hubei Univ Med. 2020;39(4):349–353+357.

[59] Yan Y, Wang H, Deng C, Zhang G, Chen Y, Shen X, Cheng H, Peng L. Effects of growth years, altitude, and light conditions on levels of eight components in rheum palmatum. Chin Herbal Med. 2017;48(11):2285–2291.

[60] Wang Q, G Du. Analysis of differential secondary metabolites in stems of dendrobium nobileunder wild-simulating stone cultivation based on metabolomics. Chin J Exp Formulae. 2024;30(10):169–175.

[61] Wu Q, P Su, Y Liu. Research progress on the antioxidant and pro-oxidative properties of flavonoids. Food Indus Technol. 2014;35(24):379–383.

[62] Shang J, W Wu, Y Ma. Metabolic pathways of phenylpropane in plants. Chin J Biochem Mol Biol. 2022;38(11):1467–1476.

[63] Li R, Guo Y, Xie C, Jia Y, Zhang B, Luo H, Wu Z. Effects of methyl jasmonate treatment on phenylpropanoid metabolism in fresh-cut muskmelon during storage. Food Indus Technol. 2023;44(15):354–361.

[64] Kim JI, Zhang X, Pascuzzi PE, Liu C, Chapple C. Glucosinolate and phenylpropanoid biosynthesis are linked by proteasome-dependent degradation of PAL. New Phytol. 2020;225(1):154–168. doi: 10.1111/nph.16108. 31408530

[65] Lei Y, Cao Y, Chen X, Li W. Determination of resveratrol content in different tissues of *Reynoutria japonica* its callus. Forest Prod Chem Indus. 2007;S1:109–112.

[66] Sun B, Shen Y, Chen S, Shi Z, Li H, Miao X. A novel transcriptional repressor complex MYB22-TOPLESS-HDAC1 promotes rice resistance to brown planthopper by repressing F3'H expression. New Phytol. 2023;239(2):720–738. doi: 10.1111/nph.18958. 37149887

[67] Li P, Li Q, Huang Y, Huang R. Research progress on plant flavonoids resistant to uv-b radiation. J Ecol. 2001;20(6):36–40.

[68] Tu L. The effects of water stress on the physiology and major secondary metabolites of *Tripterygium wilfordii*. 2019.

[69] Cai N, Dan R, Chen P. The effect of water stress on the flavonoid content of buckwheat seedlings. J Northwest Agric Sci. 2008;17(4):91–93.

[70] Ge M, Jin Z, Li J, Zhong Z, Zhang L. Preliminary study on the flavonoid composition in Sargentodoxa cuneata. J Zhejiang Forestry Univ. 2002; 4:48–52.

[71] Zhu Z, Wen D, Zhang D, Cao E, Dong G, Du W, Sun W, Krzysztof D, Shi Y, Xue J. Exploration of the molecular mechanism by which ultraviolet light promotes the accumulation of flavonoids in bitter buckwheat. Chin Herbal Med. 2021;52(5):1448–1453.

[72] Wang H, Xie B, Jiang Y, Wang M. The influence of light intensity on the development of Ginkgo biloba leaves and the contents of flavonoids and lactones. J Jiangxi Agric Univ (Nat Sci). 2002;5:617–622.

[73] Xiao L, Q Lu, R Li. Effects of light quality, light intensity, and explant type on callus proliferation and resveratrol accumulation in *Reynoutria japonica*. Jiangsu Agric Sci. 2016;44(11):60–63.

[74] Hua W, Z Wang. Cloning and sequence analysis of *Gentiana macrophyllum* 10-hydroxylase (G10H) gene. Genomics Appl Biol. 2013;32(4):510–515.

[75] Chai J, Wang X, Yi C, Zhou J, Liu D, Zhao N, He T. Preliminary screening of differentially expressed genes in the flavonoid metabolic pathway of *Polygonatum odoratum*. Mol Plant Breed. 2023:1–12.

[76] Zhu C. A brief analysis of the differences in medicinal components of different parts of root and rhizome medicinal materials. Agric Sci Technol Inf. 2024;3:14–18.

[77] Cao X. Research on the effects of nitrogen, phosphorus and potassium nutrient levels on the growth and secondary metabolites of Scutellaria baicalensis. 2012. Xianyang/Province, Shaanxi.

[78] Yu Z. Chengdu University of TCM. Research on the photosynthetic characteristics and nutrient absorption patterns of *Reynoutria japonica* on its quality formation mechanism. 2016:98.

[79] Ma Y, D Wan, Q Huang. Correlation analysis of different soil factors and the main components of *Reynoutria japonica*. Shizhen Trad Chin Med Chin Herbal Med. 2009;20(6):1520–1522.

[80] Nicolas-Espinosa J, Garcia-Ibañez P, Lopez-Zaplana A, Yepes-Molina L, Albaladejo-Marico L, Carvajal M. Confronting secondary metabolites with water uptake and transport in plants under abiotic stress. Int J Mol Sci. 2023;24(3):2826. doi: 10.3390/ijms24032826. 36769147 PMC9917477

[81] Medina-Bolivar F, Condori J, Rimando AM, Hubstenberger J, Shelton K, O’Keefe SF, Bennett S, Dolan MC. Production and secretion of resveratrol in hairy root cultures of peanut. Phytochemistry. 2007;68(14):1992–2003. doi: 10.1016/j.phytochem.2007.04.039. 17574636

[82] Liu Z-P, Tang W, Wang G, Xu J, Zhu L, Lyu Q, Wang W, Chen X, Ding W. Ferulic acid inhibiting colon cancer cells at different Duke’s stages. Food Med Homol. 2025;2(3), 9420063.

[83] Zhang F, Wu R, Liu Y, Dai S, Xue X, Li Y, Gong X. The food and medicine homologous chinese medicine from leguminosae species: a comprehensive review on bioactive constituents with neuroprotective effects on nervous system. Food Med Homol. 2025;2(2), 9420033.

[84] Zhang F, Wu R, Liu Y, Dai S, ue X X, Li Y, Gong X, et al. Nephroprotective and nephrotoxic effects of rhubarb and their molecular mechanisms. Biomed Pharmacother. 2023;160: 114297. doi: 10.1016/j.biopha.2023.114297. 36716659

[85] Kang L, Li D, Jiang X, Zhang Y, Pan M, Hu Y, Si L, Zhang Y, Huang J, et al. Hepatotoxicity of the major anthraquinones derived from polygoni multiflori radix based on bile acid homeostasis. Front Pharmacol. 2022;13: 878817. doi: 10.3389/fphar.2022.878817. 35662717 PMC9157432

[86] Alum EU, Obasi DC, Abba JN, Aniokete UC, Okoroh PN, Paul-Chima Ugwu O, Uti DE, et al. Endogenous plant signals and human health: molecular mechanisms, ecological functions, and therapeutic prospects. Biochem Biophys Rep. 2025;43: 102114. doi: 10.1016/j.bbrep.2025.102114. 40678797 PMC12268104

[87] Ren ZQ, Zheng SY, Sun Z, Luo Y, Wang YT, Yi P, Li YS, Huang C, Xiao WF, et al. Resveratrol: molecular mechanisms, health benefits, and potential adverse effects. MedComm (2020). 2025;6(6), e70252. doi: 10.1002/mco2.70252. 40502812 PMC12152427

[88] Alum EU, Udechukwu CD, Obasi DC. RNA signaling in medicinal plants: An overlooked mechanism for phytochemical regulation. Biochem Biophys Rep. 2025;42: 102032. doi: 10.1016/j.bbrep.2025.102032. 40342531 PMC12059694

